# Antibacterial effects of platelet-rich fibrin produced by horizontal centrifugation

**DOI:** 10.1038/s41368-020-00099-w

**Published:** 2020-11-26

**Authors:** Mengge Feng, Yulan Wang, Peng Zhang, Qin Zhao, Shimin Yu, Kailun Shen, Richard J. Miron, Yufeng Zhang

**Affiliations:** 1 State Key Laboratory Breeding Base of Basic Science of Stomatology (Hubei-MOST) and Key Laboratory of Oral Biomedicine, Ministry of Education, School and Hospital of Stomatology, Wuhan University, Wuhan, China; 2 Department of Periodontology, School of Dental Medicine, University of Bern, Bern, Switzerland; 3 Medical Research Institute, School of Medicine, Wuhan University, Wuhan, China

**Keywords:** Bacterial techniques and applications, Bacterial infection

## Abstract

Platelet-rich fibrin (PRF) has been widely used owing to its ability to stimulate tissue regeneration. To date, few studies have described the antibacterial properties of PRF. Previously, PRF prepared by horizontal centrifugation (H-PRF) was shown to contain more immune cells than leukocyte- and platelet-rich fibrin (L-PRF). This study aimed to compare the antimicrobial effects of PRFs against *Staphylococcus aureus* and *Escherichia coli* in vitro and to determine whether the antibacterial effects correlated with the number of immune cells. Blood samples were obtained from eight healthy donors to prepare L-PRF and H-PRF. The sizes and weights of L-PRF and H-PRF were first evaluated, and their antibacterial effects against *S. aureus* and *E. coli* were then tested in vitro using the inhibition ring and plate-counting test methods. Flow-cytometric analysis of the cell components of L-PRF and H-PRF was also performed. No significant differences in size or weight were observed between the L-PRF and H-PRF groups. The H-PRF group contained more leukocytes than the L-PRF group. While both PRFs had notable antimicrobial activity against *S. aureus* and *E. coli*, H-PRF demonstrated a significantly better antibacterial effect than L-PRF. Furthermore, the antimicrobial ability of the PRF solid was less efficient than that of wet PRF. In conclusion, H-PRF exhibited better antibacterial activity than L-PRF, which might have been attributed to having more immune cells.

## Introduction

Dental implants are increasingly accepted by patients with missing teeth, with high survival and success rates^1^. However, the loss of bone and soft tissue often limits dental implant placement. In such cases, clinicians perform soft tissue transplantation, guided bone regeneration (GBR), or sinus augmentation to address these limitations. Although most of these techniques provide predictable results, improvements in wound healing and bone and soft tissue regeneration are needed both after tooth extraction and during implant placement. Recently, second-generation platelet-rich fibrin (PRF) was proposed as a new implant therapeutic strategy for promoting implant healing and bone and soft tissue integration^2,3^. PRF or leukocyte- and platelet-rich fibrin (L-PRF) is obtained from the inpatients’ blood and typically centrifuged at a relative centrifugal field (RCF)-max/g-force of 700 for 12 min without any additives^4,5^. PRF not only acts as a three-dimensional fibrin scaffold but also contains numerous autologous cells, such as platelets, macrophages, and neutrophils^6^. Furthermore, the fibrin matrix of PRF serves as a “storage” scaffold for the gradual release of growth factors over time^7^. Given its conformance to the criteria for tissue engineering, PRF has been widely used in dentistry, showing great therapeutic potential for both soft and hard tissue regeneration^4,8,9^.

However, considering that the oral cavity harbors hundreds of microbes^10,11^, wounds healing after tissue regeneration are always at risk of infection. Based on previous reports, *Staphylococcus aureus and Escherichia coli* are related to these infections^12–14^. For patients subjected to implant surgery, this infection often leads to the inflammation of soft tissue around the implant and progressive bone loss, resulting in implant loss^15^. In anti-infection immunity, immune cells play an important role by acting as a bodily defense mechanism. PRF contains numerous immune cells, which may inhibit the infection associated with implant placement. Although several studies have reported that PRF has an antibacterial effect, there are no reports comparing the antibacterial effects of PRFs prepared using different centrifugation methods or protocols. Furthermore, whether the antibacterial effect of PRF is correlated with the number of immune cells present in the final composition remains unexplored^16,17^.

Recently, horizontal centrifugation of PRF was shown to result in better cell layer separation and to minimize cell accumulation on the distal surfaces of centrifugation tubes, which prevents proper cell layer separation^5,18–20^. In horizontal centrifugation, the swing-out bucket produces a completely horizontal tube. This can result in substantial differences between the minimum and maximum centrifugal radii of the tube, which causes differences between the RCF-min and RCF-max, respectively. Furthermore, we previously demonstrated that horizontal centrifugation led to a fourfold increase in immune cell numbers when compared to those achieved with fixed-angle centrifugation^5^. Considering that platelets and leukocytes are crucial in immune defense against infection, PRF obtained by horizontal centrifugation (H-PRF) may exert greater antibacterial effects than PRF or L-PRF.

This study aimed to accomplish the following: to compare the antimicrobial effects of PRF prepared by horizontal centrifugation (H-PRF) and L-PRF produced on a fixed-angle centrifuge against *Staphylococcus aureus* and *Escherichia coli* in vitro and to determine whether the antibacterial effects were correlated with the immune cell numbers in H-PRF and L-PRF.

## Results

### Preparation of L-PRF and H-PRF

**Fig. 1:**
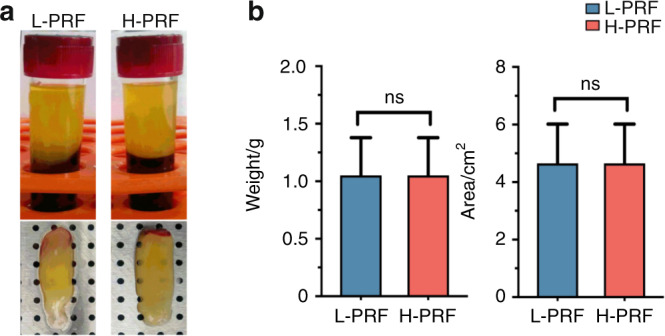
Photos of leukocyte- and platelet-rich fibrin (L-PRF) and H-PRF. **a** Photos of platelet-rich fibrin (PRF) obtained after centrifugation by both protocols. Note the horizontal-layer centrifugation in H-PRF versus the angular centrifugation in L-PRF. **b** Weight and size measurements of PRF clots

After centrifugation, the blood samples were separated into various layers. Using fixed-angle centrifugation, an angular red blood layer was observed following L-PRF preparation, whereas a horizontal division between the PRF and red blood cell layers was found in the H-PRF group prepared by horizontal centrifugation (Fig. 1a). No significant differences in size or weight were observed between the L-PRF and H-PRF groups when both groups were centrifuged in 10 mL glass tubes (*P* > 0.05, Fig. 1b).

### Antibacterial properties of L-PRF and H-PRF

**Fig. 2:**
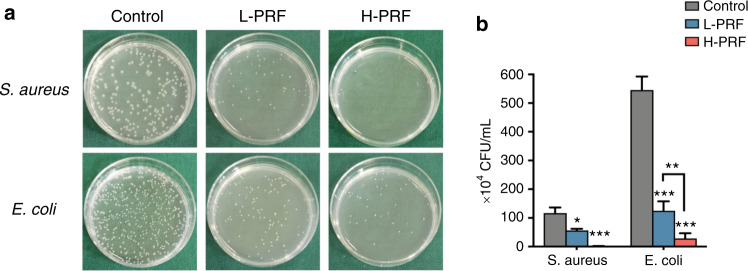
Antibacterial properties of leukocyte- and platelet-rich fibrin (L-PRF) and H-PRF. **a**, **b** Photos and quantitative analysis of *S. aureus* and *E. coli* bacterial colonies incubated with L-PRF or H-PRF clots for 4 h. *P < 0.05, **P < 0.01, and ***P < 0.001

The colony-forming unit (CFU) measurements indicated that both PRF clots had antibacterial effects against *S. aureus* and *E. coli* (Fig. 2a, b). Compared with L-PRF, H-PRF showed a significant and pronounced increase in activity against both *S. aureus* and *E. coli*.

**Fig. 3:**
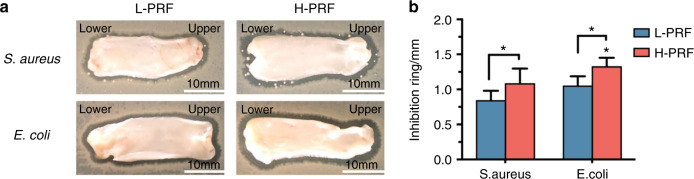
Antibacterial effects of leukocyte- and platelet-rich fibrin (L-PRF) and H-PRF. **a**, **b** Photos and quantification of the inhibition zones of L-PRF and H-PRF membranes incubated with *S. aureus* or *E. coli* for 24 h. *P < 0.05, **P < 0.01, and ***P < 0.001

Next, the antibacterial activities of the PRF clots were analyzed by direct incubation on bacterial culture plates. After 24 h of incubation, the antimicrobial activity was demonstrated by investigating the clear zones from the inhibition rings around the L-PRF and H-PRF groups (Fig. 3a). The width of the inhibition zone in the H-PRF group was significantly larger than that in the L-PRF group for both bacteria. Furthermore, the inhibition zone against *E. coli* was wider than that against *S. aureus* (*P* < 0.05, Fig. 3b).

### Antibacterial properties of different layers from L-PRF and H-PRF

**Fig. 4:**
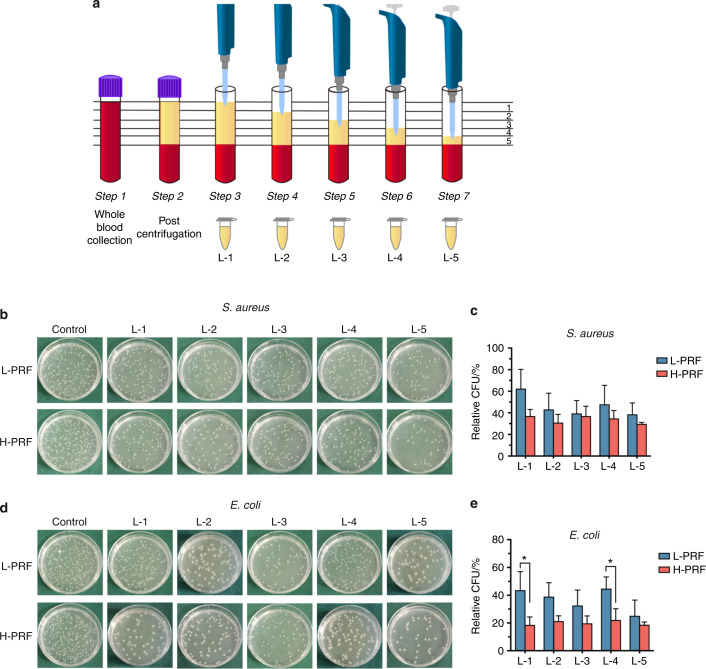
Antibacterial properties of different layers of platelet-rich fibrin (PRF) prepared in accordance with leukocyte- and platelet-rich fibrin (L-PRF) and H-PRF protocols. **a** Illustration of the stratified sampling procedure. PRF was equally divided into five layers. **b**, **c** Photos and quantitative analysis of the *S. aureus* bacterial colony incubated with each L-PRF or H-PRF layer for 4 h. **d**, **e** Photos and quantitative analysis of the *E. coli* bacterial colony incubated with each L-PRF or H-PRF layer for 4 h. *P < 0.05, **P < 0.01, and ***P < 0.001

Previously, we observed that the cell contents in each PRF layer were different, with H-PRF harboring up to four times more leukocytes than L-PRF^5^. Therefore, we divided the liquid-state PRF into five equal portions after centrifugation in plastic tubes to verify whether the antibacterial properties were different in each layer (Fig. 4a). The plate-counting assay showed that each PRF layer exhibited some antibacterial activity against *S. aureus* and *E. coli*. However, the antimicrobial effects of the five layers of each PRF varied significantly. Our CFU results showed that the bacterial count of layer five in L-PRF was the lowest among all those analyzed. However, there were no significant differences in the antibacterial effects of the different H-PRF layers on the growth of *S. aureus* and *E. coli* (Fig. 4b–e).

### Flow-cytometric analysis

**Fig. 5:**
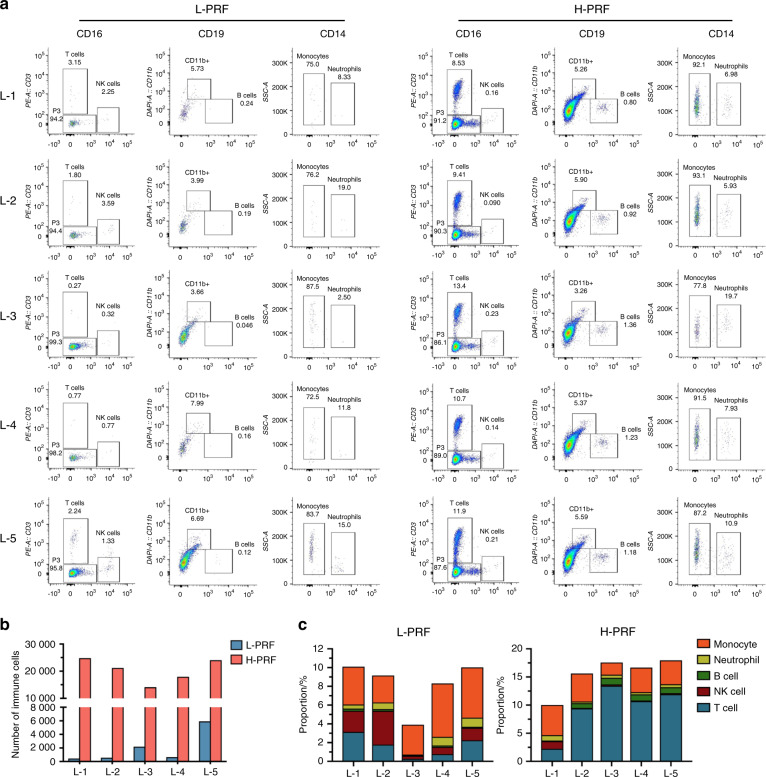
Flow-cytometric analysis of immune cells in each layer of leukocyte- and platelet-rich fibrin (L-PRF) and H-PRF. **a** Flow-cytometric profiles of different layers stained for immune cells (CD45 + ), T cells (CD3 + ), NK cells (CD16 + ), B cells (CD19 + ), neutrophils (CD11b + ; CD14 + ), and monocytes (CD11b + ; CD14−). **b** Quantitative analysis of the total number of immune cells in each L-PRF and H-PRF layer. **c** The percentages of T cells, NK cells, B cells, neutrophils, and monocytes among total immune cells in each L-PRF and H-PRF layer

Figure 5a and Table 1 show the gates and numbers of various immune cells from various layers. In general, the number of total immune cells in H-PRF was tenfold higher than that in L-PRF (102,076 vs. 9778), particularly in the upper layers (Fig. 5b). For L-PRF, the majority of immune cells were located only within the fifth layer nearest to the buffy coat, whereas in the H-PRF group, the number of immune cells was increased in all layers but also more evenly distributed throughout the upper layers (Fig. 5b and Table 1). The proportions of immune cell subtypes were different in each layer in the L-PRF and H-PRF groups (Fig. 5c).

**Table 1:** Cell counts of L-PRF and H-PRF by flow-cytometric analysis

Group	Layer	Immune cells	T cell	NK cell	B cell	Neutrophil	Monocyte
L-PRF	L-1	445	14	10	1	2	18
	L-2	557	10	20	1	4	16
	L-3	2 200	6	7	1	2	70
	L-4	650	5	5	1	6	37
	L-5	5 926	133	79	7	57	318
H-PRF	L-1	24 777	2 114	39	181	83	1 095
	L-2	21 191	1 995	19	176	67	1 051
	L-3	14 109	1 896	33	165	78	308
	L-4	17 940	1 917	25	196	68	785
	L-5	24 059	2 870	51	249	128	1 026

### Antibacterial effects of PRF solid and exudate components from each layer in L-PRF and H-PRF

**Fig. 6:**
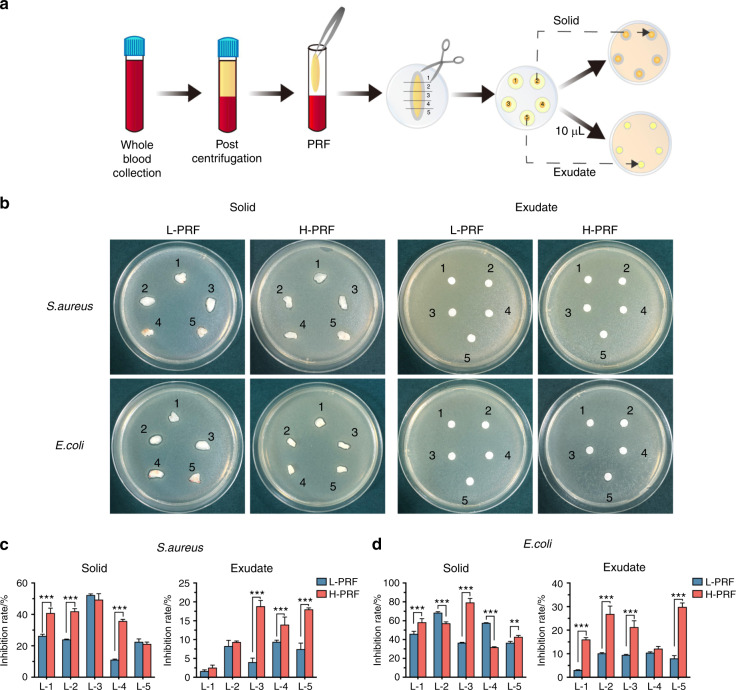
The antibacterial properties of solid and liquid layers obtained via leukocyte- and platelet-rich fibrin (L-PRF) and H-PRF protocols. **a** Illustration of the stratified sampling and incubation procedure. The total platelet-rich fibrin (PRF) clot was divided into five equal portions and then separated into solid and exudate components. **b**–**d** Photos and quantitative analysis of the inhibition zones of each L-PRF and H-PRF layer against *S. aureus* and *E. coli* after incubation for 24 h. *P < 0.05, **P < 0.01, and ***P < 0.001

PRF clots were then separated into either solid or exudate components and further subjected to antimicrobial assays (Fig. 6a). The solid and exudate components from L-PRF and H-PRF had different antibacterial properties (Fig. 6b–d). Consistent with previous results, the antibacterial effects of the solid and exudate components against *E. coli* were better than those against *S. aureus*. Furthermore, the relative inhibition rates of H-PRF exudates were significantly better than those of L-PRF, particularly for *E. coli* (Fig. 6c, d).

## Discussion

Recently, PRF has been considered as a potential strategy for promoting tissue regeneration around implants^2^. However, implants surrounding tissues are exposed to various bacterial infiltrations; hence, strategies to combat bacterial overload are needed. In the existing literature, only a few reports have discussed the antibacterial effects of PRF, especially when compared to the number of studies investigating its regenerative potential. PRF is a complex mixture of cells (platelets and various white blood cells), a three-dimensional fibrin scaffold and a variety of active growth factors and plasma proteins^21^. However, the antibacterial properties of PRF are relatively unknown. Therefore, this study aimed to compare the antimicrobial activities of PRFs prepared via fixed-angle (L-PRF) and horizontal (H-PRF) centrifugation against Gram-positive and Gram-negative bacteria and to determine whether the antibacterial effects were correlated with the number of immune cells.

It is well-known that PRFs contain accumulated platelets. Platelets not only generate oxygen metabolites and antimicrobial peptides directly targeting bacterial cells but also play a role in the binding, aggregation, and internalization of pathogens to improve clearance via the bloodstream^22–24^. Burnouf et al.^16^ reported that platelet concentrates inhibited the growth of *S. aureus*, *E. coli*, *Pseudomonas aeruginosa*, and *Klebsiella pneumonia*. In addition, platelet gel supernatants have been shown to exert bactericidal effects against *S. aureus*^25^. In contrast, leukocytes are well-known white blood cells that have a considerable bactericidal ability. Upon bodily infection, activated neutrophils migrate to the infection site and release active antibacterial substances to promote the phagocytosis of foreign pathogens. Leukocytes produce a variety of antimicrobial peptides and enzymes, including lactoferrin, defensins, BPI azurocidin/heparin-binding protein, cathelicidins, phospholipase A2, and calprotectin^26^. Moreover, plasma contains a complement system, which can activate the complement cascade to facilitate bacterial cell lysis and leukocyte recruitment for humoural defense against infectious agents^27^.

We used the classic inhibition ring test and plate-counting test to verify the antibacterial effect according to a previous report^28–30^. Our results indicated that L-PRF and H-PRF exerted certain antibacterial effects against *S. aureus* and *E. coli*. However, compared with L-PRF, H-PRF showed a significantly better ability to protect against *E. coli* regardless of whether it was in the supernatant or gel (fibrin) state. Although the majority of blood platelets and leukocytes remain within the PRF membranes, the exudate also contains small amounts of cellular components (2.5% of platelets and 0.9% of leukocytes)^31^.

Previous research has shown that the platelets and leukocytes in PRFs obtained by horizontal centrifugation accumulate cells up to four times more effective than those obtained by fixed-angle centrifugation. In our study, flow-cytometric analysis provided more accurate data regarding immune cell types, which were obtained by centrifugation, and further confirmed that the total immune cell numbers were increased almost tenfold in H-PRF compared with L-PRF. Researchers have reported that the antibacterial properties of PRF can be divided into two parts: (1) the release of antimicrobial peptides entrapped initially in the fibrin matrix along with PRF degradation and (2) the antimicrobial factors produced constantly from the cells inside the fibrin matrix^28^. The increased leukocyte counts within H-PRF were hypothesized to dramatically enhance the antimicrobial properties of PRF compared with those achieved with traditional L-PRF protocols. This finding was also supported by Cieślik-Bielecka et al.^32^.

Previously, using complete blood count analysis, the cell content in each upper layer of PRF was shown to differ^5^. We divided the PRFs into five equal portions to investigate the differences in immune cell numbers and their antibacterial effects and to explore whether the antibacterial properties correlated with these cell numbers. The upper layer of L-PRF had the fewest immune cell numbers, which correlated well with the antibacterial properties of that layer and further demonstrated weaker activity when compared with that of the other layers. Although the numbers of immune cells in L-1 and L-5 from H-PRF were nearly the same, the antibacterial effect of L-5 was increased, particularly in the exudate. Flow-cytometric analysis showed that the cell proportions were significantly different between L-1 and L-5, probably because various white blood cells had different cell densities. The fifth layer of H-PRF contained more T cells than the first layer. T cells are directly implicated in the elimination of microbial pathogens by releasing cytotoxic granules and bacteriostatic or lytic molecules^33,34^. Moreover, T cells can regulate other immune cells, including dendritic cells, macrophages, B cells and other subtypes of T cells^35–37^. We attributed the improved antibacterial ability of L-5 to the good regulatory effects of T cells on other immune cells found in the layer closest to the buffy coat in H-PRF. Furthermore, both the solid-membrane component and the exudate from H-PRF were antibacterial, which indicated that not only the cell components of H-PRF but also the secretions of cytokines and/or complement proteins had strong antimicrobial properties. Although we found that the antibacterial effect of PRF is related to the number of immune cells, the exact mechanisms have not been elucidated, and further experiments must be conducted in the future.

## Conclusion

Our study demonstrated that the PRF prepared by horizontal centrifugation exhibited significantly better antibacterial activities against both *S. aureus* and *E. coli* than traditional L-PRF. The increased antibacterial effects of H-PRF were not only attributed to the increase in leukocytes but also correlated with released exudate components. The antimicrobial ability of the PRF solid was less efficient than that of wet PRF, which indicated that the liquid components of PRF could be retained to enhance antibacterial properties during routine clinical use.

## Materials and methods

### Preparation of PRF

Blood samples were collected from eight volunteers, including three males and five females (average age of 25) after informed consent was provided. All the protocols used in this study were approved by the Ethics Committee of the School and Hospital of Stomatology, Wuhan University (B52/2020). All participants were in good health, were nonsmokers, had no symptoms of infection, and had taken no antibiotics for at least 3 months prior to the experiments.

In this study, the two types of PRF were prepared with two different centrifugation devices. In the present study, the plastic tubes were used to obtain the liquid form of PRF, while glass tubes were used to obtain the solid form of PRF according to previous reports^4,5,38^. L-PRF was obtained by a fixed-angled centrifuge (Chixin Biotech, Wuhan, China) at a g-force of 700 for 12 min at room temperature according to previous reports^4,5,38^. H-PRF was collected by a horizontal centrifuge at a g-force of 700 for 8 min at room temperature according to a previous report^5^.

### PRF size and weight

L-PRF and H-PRF clots were obtained by centrifugation in 10-mL glass tubes (Chixin Biotech, Wuhan, China). After centrifugation, the red blood clots attached to the yellow PRF clots were gently removed^6,39^. Each PRF clot was measured with a Vernier caliper and weighed^40^. All paired L-PRF and H-PRF samples used in the experiment were obtained from the same donors.

### Bacterial preparation

*S. aureus* (ATCC BAA-1758) and *E. coli* (MG 1655) were grown in LB broth at 37 °C under aerophilic conditions. The optical density at 600 nm was measured by a microplate reader (PowerWave XS2; BioTek, Winooski, VT). The bacterial suspension was diluted to 1 × 10^6^ or 1 × 10^5^ CFU·mL^−1^ for experimental use.

### Inhibition ring test of the whole PRF clot

L-PRF and H-PRF clots were obtained by centrifugation in 10-mL glass tubes. L-PRF and H-PRF clots were compressed and converted into a standardized membrane with a thickness of 1 mm to determine their antibacterial abilities^28^. A total of 500 μL of *S. aureus* or *E. coli* (1 × 10^6^ CFU· mL^−1^) was cultured on LB agar plates. The L-PRF or H-PRF membranes on the surface of the LB agar plate were in direct contact with *S. aureus* or *E. coli.* The samples were incubated for 24 h. The length and width of each PRF membrane were measured at baseline after 24 h using ImageJ. Horizontal (length) and vertical (width) lines were drawn at a 90° angle from the midpoint of the membrane^28^.

### Inhibition ring test of the PRF exudate and solid PRF

The L-PRF and H-PRF clots were obtained by centrifuging the blood in 10-mL glass tubes. The obtained L-PRF and H-PRF clots were first divided into five equal parts to compare the antibacterial properties of the different PRF layers. Then, each PRF clot layer was drained into the exudate and solid parts (containing nearly no exudate). Ten microlitres of exudate from each clot were pipetted onto the filter paper^29^. Each drained L-PRF and H-PRF clot layer was then compressed and converted into a 1-mm-thick membrane. A total of 500 μL of *S. aureus* or *E. coli* (1 × 10^6^ CFU· mL^−1^) was cultured on LB agar plates. After 30 min, the PRF membrane or filter paper was placed directly on the surface of the LB agar plate. The incubation time was also 24 h.

The length and width of each PRF membrane or filter paper were measured by ImageJ. The calculation procedure was the same as that described above.

### Plate-counting test of the whole PRF clot

L-PRF and H-PRF clots were obtained by centrifugation in 10-mL glass tubes. L-PRF and H-PRF clots were mixed with 4 mL of bacteria (1 × 10^5^ CFU· mL^−1^) using a shaking incubator for 4 h (37 °C, 150 r·min^−1^) to investigate their antibacterial properties. Afterward, 100 µL of the sample was diluted with 900 µL of phosphate-buffered saline (PBS), and 30 μL of the sample was plated onto an LB agar plate at 37 °C overnight for CFU counting^30^.

### Plate-counting test of the liquid form of PRF

The liquid forms of L-PRF and H-PRF were obtained by centrifugation in plastic tubes (Chixin Biotech, Wuhan, China) to detect the antibacterial properties of each layer. Then, the different layers were partitioned by sequentially pipetting 870 μL (±10 μL) from the top layer down to the red blood cell interface (not pipetting the red blood cell layer). One hundred microlitres of L-PRF (1-5), H-PRF (1-5), or PBS were mixed with 100 µL of *S. aureus* or *E. coli* (1 × 10^6^ CFU· mL^−1^) in 1.5-mL sterile EP tubes. A final 1 mL sample in PBS was obtained at a final bacterial concentration of 1 × 10^5^ CFU· mL^−1^. After incubating the tubes at 37 °C at 150 r·min^−1^ for 4 h, 100 µL of the sample was collected and diluted with 900 µL of PBS. Then, 30 µL of each sample was added to an LB agar plate and cultured at 37 °C overnight for CFU counting^41,42^.

### Preparation of single-cell suspensions and flow-cytometric analysis

The L-PRF and H-PRF layers were obtained by centrifugation in 10-mL plastic tubes and divided into five equal portions. Cells from each layer were then centrifuged. Afterward, the cells were resuspended and incubated for 30 min on ice with FITC anti-human CD45 (1:200, Biolegend, no. 304006), PE anti-human CD3 (1:200, Biolegend, no. 300308), Pacific Blue™ anti-mouse/human CD11b (1:200, Biolegend, no. 101224), Alexa Fluor^®^ 700 anti-human CD14 (1:200, Biolegend, no. 325614), PE/Cy7 anti-human CD16 (1:200, Biolegend, no. 302015) and APC anti-human CD19 (1:200, Biolegend, no.302212) antibodies to quantify the various blood cell types. Finally, flow-cytometric analysis was performed using a BD LSRFortessa instrument (USA), and the results were analyzed by FlowJo 10.

### Statistical analysis

The data were analyzed with GraphPad Prism software 7.0 (La Jolla, CA). The *t* test was used to determine statistical significance. Data are reported as the mean ± SD. *P < 0.05, **P < 0.01, and ***P < 0.001 considered statistically significant.
